# *Tamarix arabica* and *Salvadora persica* as antibacterial agents

**DOI:** 10.3934/microbiol.2020008

**Published:** 2020-05-18

**Authors:** Awatif A. Al-Judaibi

**Affiliations:** Department of Biological Sciences-Microbiology section, Faculty of Science, Jeddah University/King Abdulaziz University, Jeddah, Saudi Arabia, P.O. Box: 13520, Jeddah 21414, Saudi Arabia

**Keywords:** Novel EC plate, *Tamarix Arabica*, *Salvadora persica*, kill-time, ion leakage, GC mass

## Abstract

Despite the harsh conditions and limited water resources of the Arabian Peninsula, plants that live in this environment contain a variety of bioactive compounds and have been used in traditional medicines for thousands of years. We investigated the effects of ethanol extracts of *Tamarix arabica* and *Salvadora persica* on Gram-negative and Gram-positive bacteria. The investigations were include; the inhibition of the bacterial growth, determination of MIC and MBC, detection of kill-time, potassium and phosphorus leakages and detection of the bioactive compounds by the GC-MS analysis. The tested extracts in combination, at a 1:1 volume ratio, showed high inhibitory effects, as reflected by the minimum inhibitory concentrations and minimum bactericidal concentrations. The new EC plate was used to determined MBC and kill-time. Further, the detection of phosphate and potassium leakage indicated a loss of selective permeability of the cytoplasmic membrane after treatment with these extracts. The bioactive compounds in the ethanol extracts of *T. arabica* and *S. persica* may offer a less expensive and natural alternative to pharmaceuticals.

## 1. Introduction

The random use of antibiotics has led to the development of new bacterial strains with the ability to resist antibiotic treatment, resulting in the loss of efficacy of many antibiotics currently on the market. Antibiotic-resistant bacteria (ARB) and antibiotic resistance genes (ARGs) have become widespread in wastewater and irrigation water and therefore, they can be transferred to crops and vegetables, and subsequently, to humans. Alternatively, ARB and ARGs may be transferred to animals and then to humans. Several studies have found that ARB and ARGs are propagated by agricultural irrigation and the treatment of wastewater [1]–[5]. Hocquet et al. [6] presented data on antibiotic-resistant strains of *Escherichia coli* and *Pseudomonas aeruginosa*, which have extended-spectrum β-lactamases as their ARGs, and they concluded that antibacterial resistance is an ‘environmental pollutant’. A study of antibiotic resistance bacteria in China found that ARGs are highly prevalent and diverse in soils, plant effluent, sewage treatment, surface water, and animal waste, due to the use of antibiotics [7]. As a result of these changes in bacterial responses to antibiotics, the investigation of new sources of antibiotics, including medicinal plants, is required. Thus, many studies have investigated the antimicrobial properties of active compounds from medicinal plants, sea animals, and some microorganisms. Antimicrobial studies of medicinal plants have shown that these plants exhibit bioactivity against microorganisms and can be effective at treating diseases, such as intestinal disorders, diarrhea, colitis, and digestive problems, including flatulence, stomach ache, and indigestion [8]. In a study on the potential antimicrobial activity of green tea polyphenols and *Triphala* against *Enterococcus* faecalis, significant antibacterial activity was demonstrated, and these medicinal plants could be used to improve irrigation treatment of the root canal [9].

In a study of the effects of alcoholic, etheric, and aqueous extracts of *Magnolia grandiflora* L., *Melissa officinalis* L., *Thymus vulgaris* L., and *Rhus corriaria* L. against *Streptococcus mutans* L. and *S. sanguis* L., antibacterial activities of the ethanol extract of *M. officinalis* were observed against both bacterial strains [10].

Alcohol extracts of *Achillea crithmifolia*, *A. grandifolia*, *Angelica pancicii*, *A. sylvestris*, *Artemisia absinthium*, *Laserpitium latifolium*, *Tanacetum parthenium*, *Cynodon dactylon*, *Curculigo orchioides*, *Cinnamomum camphora*, *Curcuma longa*, *Maesa lanceolata*, *Calpurnia aurea*, *Elaeodendron croceum*, *Hypericum roeperianum*, *Abelmoschus esculentus*, *Brassica oleracea*, *Rosa brunonii*, *Sueda fruticosa*, *Calligonum polygonoides*, *Peganum harmala* (L.), *Cucumus sativus*, *Helianthu annus*, *Melia azedarach* L., *Acacia arabica*, and *Tamarix aphylla* L. show antimicrobial activity against *Acinetobacter* sp., *Klebsiella* sp., *Proteus mirabilis*, *P. aeruginosa*, *E. coli*, *Staphylococcus aureus*, *Streptococcus pyogenes*, *S. pneumoniae*, *E. faecalis*, *Salmonella typhi*, *S. typhimurium*, *Bacillus cereus* and *B. subtilis*, with active compounds including flavonoids and polyphenols [11]–[14]. Herbal medicines are used by 80% of the world's population. The most popular herbal medicines are *Thymus vulgaris*, guava, *Glycyrrhiza glabra*, *Capsicum annum*, *Aloe vera*, papaya, turmeric, *Ocimum gratissimum*, and *Zingiber officinale* [15]–[17]. The flora of the Arabian Peninsula consists of only a few different plant species. These plants have adapted to the harsh environment and in such an environment, plants are characterized by unique bioactive compounds that classify them as medicinal plants. These plants have been used for thousands of years in traditional medicines for the treatment of diarrhea; intestinal disorders; colitis; digestive problems, including flatulence, indigestion, and stomachache; and bronchitis [14],[15],[18]–[20]. Further, medicinal plants can be used for protection against pathogenic dental biofilms of cavity-causing bacteria, including actinomycetes, *Actinobacillus*, *E. faecalis*, *Fusobacterium*, *Lactobacillus*, *Streptococcus mutans*, *Streptococcus sanguis*, *Prevotella*, and *Prophyromonas gingivalis* [21].

Medicinal plants from the Arabian Peninsula include *Rhazya stricta, Citrullus colocynthis*, mountain germander, *Pulicaria undulate, Artemisia herba-alba*, *Acacia arabica*, desert thorn, and *Ziziphus spina-christi.* These plants have shown high levels of antimicrobial activity against *S. aureus, E. coli, P. aeruginosa, Proteus vulgaris*, and *Klebsiella pneumoniae* [22]–[24]. *Tamarix arabica* and *Salvadora persica* have been studied as medicinal plants and they have been found to contain the following bioactive compounds: phosphorus, calcium, minerals, fluoride, polyphenolics, flavonoids, phenolic compounds, glycosides, terpenes, sterols, and alkaloids [25],[26].

This study was designed to investigate the antibacterial activity of alcohol extracts of *T. arabica* and *S. persica* individually and in combination as a 1:1 volume ratio. The minimum inhibitory concentration (MIC) and the minimum bactericidal concentration (MBC) were determined and the effect of the plant extracts on phosphorus and potassium leakage was assessed as an indicator of cytoplasmic membrane permeability. Furthermore, gas chromatography (GC)-mass spectrometry (MS) was performed to identify the bioactive compounds.

## 2. Materials and methods

### 2.1. Test organisms

Fast-growing, antibiotic-resistant strains of bacteria were obtained from the American Type Culture Collection (ATCC; Manassas, VA, USA). The Gram-positive bacteria included in the study were *Staphylococcus epidermidis* ATCC 12228, *S. aureus* (MRSA) ATCC 33591, *S. saparlyticus* ATCC 15305, *Streptococcus pyogenes* ATCC 19615, *S. agaloctiae* (group B) ATCC 12386, and *Enterococcus faecalis* ATCC 29212. The Gram-negative bacteria included in the study were *Senoterophomonas maltophilla* ATCC 51331, *Shigella sonnei* ATCC 9290, *Salmonella typhimurium* ATCC 14028, *Proteus vulgaris* ATCC 33420, *P. mirabilis* ATCC 35659, *Klebsilia pneumoniae* ATCC 13883, *Campylobacter jejuni* ATCC 33291, *Nisseria gonorrhoeae* ATCC 31426, *P. aeruginosa* ATCC 27853, *Enterobacter aerogenes* ATCC 29751, *Escherichia coli* ATCC 8239, *Hoemophilus influenza* ATCC 49247, *Vibrio parahoemolyticus* ATCC 17802, *Enterobacter aerogenes* ATCC 13048. Under aerobic/anaerobic conditions, bacterial strains were grown in the selected media at 37 ± 2 °C for 24 h.

### 2.2. Study plants

Two folk medicinal plants from the Arabian Peninsula, *Tamarix arabica* and *Salvadora persica*, were used in this study. Both plants where collected from Al Makhwah, Saudi Arabia during fall/winter, 2014. Plants were identified at the Botany Section of the Department of Biology at King Abdulaziz University.

### 2.3. Alcohol extraction

Leaves of *T. arabica* and *S. persica* were collected, washed with distilled water, and spread in a shaded place until dry. The dried leaves were then ground into a powder. Powdered plant extracts were prepared in 100% ethanol (1:1 w/v) in a conical flask, which was shaken at 120 rpm at 30 °C for 3 d until dried. Extracts were weighed and yield was calculated relative to the weight of the initial crude extract. The extracts were then dissolved in a 1:1 volume of dimethylsulfoxide (DMSO) and stored in a closed bottle at 4 °C.

### 2.4. Antibacterial assays

The antibacterial activity of the plant extracts was determined *in vitro* against Gram-positive and Gram-negative strains. Bioactivity was measured by disc diffusion and broth dilution methods, as described by the Clinical and Laboratory Standards Institute [27],[28]. Each extract was dissolved in DMSO to a concentration of 3 µg/mL and filtered through a 0.22 µm pore filter (Millipore, Billerica, MA, USA). The antibacterial activities of each extract were investigated by disc diffusion, using filter paper discs (1 mm diameter impregnated with 100 µL of extract) that were placed on a pre-inoculated agar surface. Negative controls were prepared using the solvent only. Plates were incubated at 37 °C for 24 h and the inhibition zones around each disc were measured. All tests were performed in triplicate.

### 2.5. Determination of MICs

MIC is defined as the lowest concentration of an antimicrobial that prevents the growth of a microorganism following a specific incubation period. MICs were determined using a broth microdilution method by 96 wells plates, as described by [29],[30]. Bacterial strains were cultured at 27 °C on Mueller Hinton Agar (MHA) and then resuspended in 1 mL of Mueller Hinton Broth (MHB; CM0405; Oxoid, Cambridge, UK) to obtain a final concentration of 1 × 10^5^ colony-forming units (CFU)/mL. Each extract was serially diluted with MHB. Following incubation, the MIC was determined as the lowest concentration of each extract for which there was no visible growth compared with the control [31]. MIC values were recorded as mg/L [29], and each treatment was performed in triplicate.

### 2.6. Determination of MBCs

MBC is defined as the lowest concentration of an antibacterial agent needed to kill 99.9% of the initial inoculum. MBCs were determined by inoculating 0.1 mL from wells showing no growth in the MIC assay, onto sterile MHA [32], in an Economic (EC) plate (patent no. 4569; King Abdulaziz City for Science and Technology, Riyadh, Saudi Arabia). Each serial concentration of the extract showing no growth in the MIC assay was cultured in one EC plate and the plates were incubated at 27 °C for 24 h. The lowest concentration showing no growth of the tested bacteria was considered as the MBC. A negative control plate was included containing only medium. MBC values were recorded as mg/L [33], and each treatment was performed in triplicate.

### 2.7. Kill-time determination

Liquid cultures (1 mL) were diluted to an initial bacterial inoculum of 2–5 × 10^5^ CFU/mL in MHB containing the MIC concentrations of *T. arabica* and *S. persica* extracts. The cultures were then incubated for 0, 2, 4, 8, 12, and 24 h at 37 °C. At each time point, 50 µL aliquots of each bacterial strain were plated on one EC plate containing MHA and were incubated at 37 °C for 24 h. Visible colonies were identified using a Scan 500 colony counter (Interscience, Woburn, MA, USA). Colonies were counted as CFU/mL [34],[35], and each treatment was performed in triplicate.

### 2.8 Determination of potassium and phosphorus leakage

This experiment was performed to estimate the secondary metabolism of the tested bacterial strains by computing the proportion of potassium, and phosphorus secreted into the medium. Potassium and phosphorus ion efflux was determined according to a previously described method [36],[37]. The concentration of free potassium, and phosphorus ions in the bacterial suspension of each bacterial strain was measured after the exposure of bacterial cells to nutrient broth for 20, 60, and 100 min. The mixture was incubated at 37 °C. Three replicates of each tube were conducted. At each pre-established interval, extracellular potassium (EasyRA Medica, Bedford, USA) and phosphorus (COBAS® INTEGRA 400 Plus Analyzer, Roche, Basel, Switzerland) concentrations were measured using photometric procedures. 300 µM of Cetyl Trimethyl Ammonium Bromide (CTAB) was used as positive control for giving 100% permeabilization (Sigma-Aldrich, Merck, Darmstadt, Germany). Results were expressed as the amount of extracellular free K^+^ and PO_3_^−^ in the growth medium (µmol/mL). Each treatment was performed in triplicate.

### 2.9. GC-MS analysis

The individual ethanol extracts of *T. arabica* and *S. persica* or a combination of both (1 g) were dissolved in methanol for 48 h. This procedure was repeated twice. The extracts were filtered through a 45 µm filter and the resulting solvent was concentrated under vacuum by nitrogen purging. Concentrated samples were resuspended in 1 mL of isooctane, filtered through a 0.2 µm filter, and stored at 4 °C until GC-MS analysis.

The samples were analyzed on a GC-MS QP2010 Plus instrument (Shimadzu, Kyoto, Japan) with a standard Rtx 5-MS capillary non-polar column (Restek Corporation, Bellefonte, PA) (dimension: 30 Mts, ID: 0.25 mm, film thickness: 0.25 µm). The flow rate of the mobile phase (carrier gas: He) was set at 1.0 mL/min. For the GC component, the oven temperature increased from 60 °C to 300 °C at 10 °C/min and the injection volume was 1 µL. Samples were dissolved in isooctane and run to completion at a range of 50–650 m/z and the results were compared with data from the National institute of standards and technology (NIST) database [38]–[41]. Samples were analyzed at the Center of Excellence in Environmental Studies, King Abdul-Aziz University.

### 2.10. Statistical analysis

The microbial zone of inhibition and cell count (CFU/mL) data were collected, summarized, and tabulated. Statistical analyses were performed using the Statistical Package for the Social Sciences, version 20 (IBM, Armonk, NY, USA). The results are expressed as mean ± standard deviation (SD). Differences between samples and the homogeneity between groups were determined using an ANOVA. Results were considered significant at *P* ≤ 0.05 and highly significant at *P* ≤ 0.01.

### References

2.11.

The references in this paper were organized using EndNote version X7 (Thomson Reuters, Toronto, Canada). References are in the APA format.

## 3. Results

The development of new antibiotics is needed to control multidrug-resistant bacteria. The effects of *T. arabica*, *S. persica*, and *T. arabica*:*S. persica* extracts on bacterial growth inhibition are shown in Table 1. High antibacterial activity of the plant extracts was seen against *E. coli*, *S. typhimurium*, and *C. jejuni*. Further, the Gram-negative bacteria were more sensitive than the Gram-positive bacteria and *T. arabica* had greater antibacterial activity than *T. arabica*:*S. persica* and *S. persica*.

**Table 1.** Inhibition of bacterial growth (mm) after 24 h of incubation with 100 µL of plant extracts.

	*T. arabica*	*S. persica*	*T. arabica:S. persica*
*S. maltophilla*	13.60 ± 0.2784**	11.50 ± 0.278**	13.20 ± 0.087**
*S. epidermidis*	11.80 ± 0.2291**	11.00 ± 0.229**	11.30 ± 0.229**
*S. agaloctiae* (group B)	9.60 ± 0.2291**	9.40 ± 0.229**	9.55 ± 0.225**
*S. saparlyticus*	11.70 ± 0.1803**	11.00 ± 0.180**	11.00 ± 0.477**
*S. pyogenes*	11.36 ± 0.0434**	11.00 ± 0.044**	10.95 ± 0.115**
*S. aureus* (MRSA)	13.67 ± 0.0700**	12.50 ± 0.070**	12.70 ± 0.180**
*E. faecalis*	17.03 ± 0.0434**	17.00 ± 0.044**	17.00 ± 0.328**
*E. aerogenes*	14.27 ± 0.0473**	12.76 ± 0.047**	13.78 ± 0.180**
*E. coli*	31.43 ± 0.0625**	30.93 ± 0.062**	30.65 ± 0.180**
*S. typhimurium*	20.80 ± 5.371**	19.3 ±5.37**	20.20 ± 0.087**
*S. sonnei*	18.30 ± 0.1803**	18.00 ± 0.180**	18.00 ± 0.180**
*P. vulgaris*	15.80 ± 0.1803**	13.00 ± 0.180**	13.75 ± 0.180**
*P. mirabilis*	11.10 ± 0.1258**	10.65 ± 0.126**	11.11 ± 0.032**
*K. pneumoniae*	11.53 ± 0.0625**	11.00 ± 0.062**	11.35 ± 0.180**
*P. aeruginosa*	11.70 ± 0.1803**	10.96 ± 0.180**	10.8 ± 0.765**
*C. jejuni*	24.90 ± 0.0866**	22.80 ± 0.087**	22.68 ± 0.180**
*N. gonorrhoeae*	14.30 ± 0.2784**	13.80 ± 0.278**	13.50 ± 0.275**
*H. influenzae*	14.00 ± 0.5269**	13.88 ± 0.527**	13.00 ± 0.229**
*V. parahoemolyticus*	10.80 ± 0.2271**	10.50 ± 0.229**	10.88 ± 0.680**

**P ≤ 0.01; ^a^Values are mean ± SD, SD = standard deviation.

### 3.1. Determination of MICs

Table 2 shows the MIC values of serial dilutions of *T. arabica*, *S. persica*, and *T. arabica*:*S. persica* on microbial growth. The lowest MIC values of *T. arabica*, *S. persica* and *T. arabica*:*S. persica* extracts were seen with *E. coli*, *C. jejuni*, *S. typhimurium*, *S. sonnei*, and *E. faecalis*. The MIC values remained constant whether the bacteria strain was treated with *T. arabica*, *S. persica*, or *T. arabica*:*S. persica* extracts. These similar MIC values implied that the plant extracts were equally effective agents against these microorganisms and can be used either on their own or in combination.

**Table 2.** Minimum inhibitory concentration (mg/L) of microbial growth after 24 h of incubation with serial dilutions of plant extracts in Mueller-Hinton broth.

	*T. arabica*	*S. persica*	*T. arabica:S. persica*
*S. maltophilla*	8	16	8
*S. epidermidis*	16	16	16
*S. agaloctiae (group B)*	32	>32	32
*S. saparlyticus*	16	16	16
*S. pyogenes*	16	16	16
*S. aureus (MRSA)*	8	8	8
*E. faecalis*	4	4	8
*E. aerogenes*	8	16	8
*E. coli*	1	1	1
*S. typhimurium*	4	4	4
*S. sonnei*	4	4	8
*P. vulgaris*	8	8	16
*P. mirabilis*	16	16	16
*K. pneumoniae*	16	16	>32
*P. aeruginosa*	16	32	32
*C. jejuni*	2	2	2
*N. gonohrae*	6	8	8
*H. influenzae*	8	8	16
*V. parahoemolyticus*	32	32	>32

### 3.2. Determination of MBCs

As indicated by the MBC results in Table 3, the *T. arabica* extract was more effective than the *S. persica* and *T. arabica*:*S. persica* extracts, against *P. vulgaris*, *S. typhimurium*, *S. sonnei*, *C. jejuni*, *S. aureus* (MRSA), and *S. maltophilla*. However, *S. persica* and *T. arabica*:*S. persica* extracts were more effective against *E. coli* and *S. saparlyticus*. Meanwhile, *T. arabica*:*S. persica* was more effective against *E. aerogenes* than either extract alone.

**Table 3.** Minimum bactericidal concentration (mg/L) of microbial growth after 24 h of incubation in Mueller-Hinton agar.

	*T. arabica*	*S. persica*	*T. arabica: S. persica*
*S. maltophilla*	8	16	8
*S. epidermidis*	16	16	16
*S. agaloctiae (group B)*	32	-	>32
*S. saparlyticus*	32	16	16
*S. pyogenes*	16	16	16
*S. aureus (MRSA)*	4	8	8
*E. faecalis*	8	8	8
*E. aerogenes*	16	16	4
*E. coli*	2	1	1
*S. typhimurium*	4	8	8
*S. sonnei*	4	8	16
*P. vulgaris*	8	16	16
*P. mirabilis*	16	16	16
*K. pneumoniae*	16	16	-
*P. aeruginosa*	16	32	32
*C. jejuni*	2	4	4
*N. gonorrhoeae*	16	8	16
*H. influenzae*	16	8	32
*V. parahoemolyticus*	32	>32	-

### 3.3. Kill-time determination

**Figure 1.**
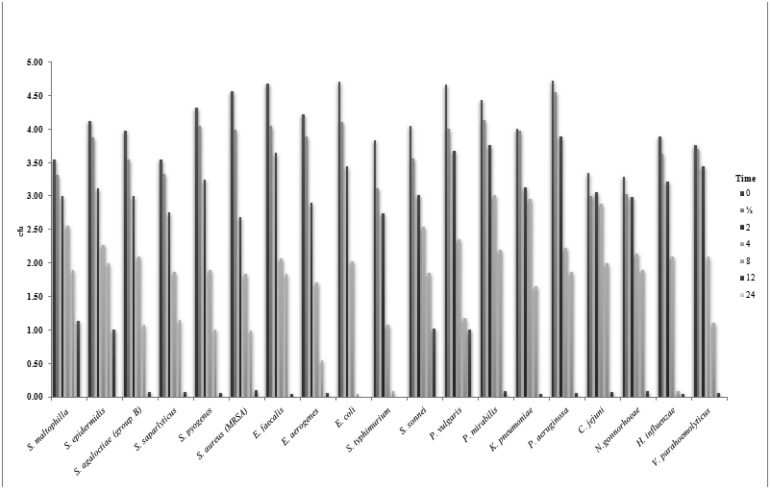
Kill-time of tested bacteria after treatment with *T. arabica* extracts for different times.

**Figure 2.**
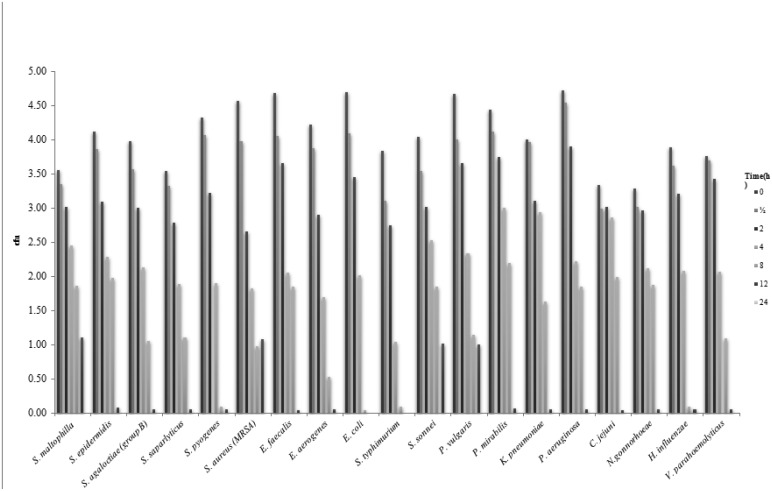
Kill-time of tested bacteria after treatment with *S. persics* extracts for different times.

**Figure 3.**
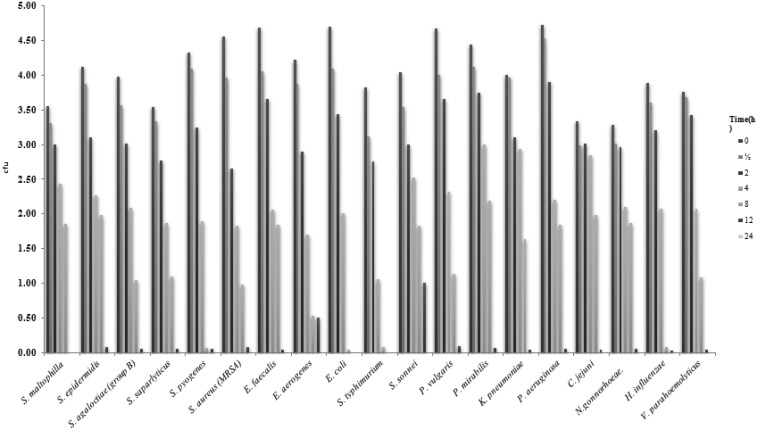
Kill-time of tested bacteria after treatment with *T. arabica:S. persics* extracts for different times.

This experiment was designed to determine the time to kill the bacterial cells after treatment with the MIC of *T. arabica*, *S. persica*, or *T. arabica*:*S. persica* extracts. The results are shown in Figures 1, 2, and 3. The kill-times for *E. coli* and *S. typhimurium* were greater than 12 h when treated with *T. arabica*, *S. persica*, and *T. arabica*:*S. persica* extracts, whereas the kill-time for all other tested strains was greater than 24 h for all extract treatments.

### 3.4. Determination of potassium and phosphorus leakage

This experiment aimed to determine the effectiveness of the plant extracts at destroying microbial cells, by measuring the amount of potassium leakage at different times of incubation. As shown in Table 4, potassium leakage from the tested bacterial strains increased with increasing incubation period with *T. arabica*, *S. persica*, and *T. arabica*:*S. persica* extracts. Moreover, a large amount of potassium leakage was observed from *E. coli, S. sonnei, P. vulgaris, K. pneumoniae, P. aeruginosa, C. jejuni, N. gonorrhoeae*, and *H. influenzae* after treatment with *T. arabica*:*S. persica*. Phosphorus leakage from Gram-positive bacteria increased with increasing incubation period, to a similar extent for all extracts (Table 5). However, the Gram-negative strains, *E. faecalis, E. aerogenes, E. coli, S. typhimurium, S. sonnei, P. vulgaris, K. pneumoniae, P. aeruginosa, C. jejuni*, and *N. gonorrhoeae*, were more affected by *T. arabica*:*S. persica* treatment than by treatment with either extract alone.

**Table 4.** Potassium leakage (mmol·l^−1^) from bacterial cells after different periods of treatment with 50 µL of plant extracts.

Incubation periods/min												
	20				60				100			
	*CTAB (-ve+ C)*	*T. arabica*	*S. persica*	*T. arabica:S. persica*	*CTAB (-ve+ C)*	*T. arabica*	*S. persica*	*T. arabica:S. persica*	*CTAB (-ve+ C)*	*T. arabica*	*S. persica*	*T. arabica:S. persica*
control	-	8.10	8.10	8.10	-	9.00	9.00	9.00	-	9.50	9.50	9.50
*S. saparlyticus*	11.03	10.45 ± 0.115**	10.32 ± 0.120**	10.34 ±.110**	11.93	10.60 ± 0.120**	10.62 ± 0.111**	10.63 ± 0.125**	12.00	10.69 ± 0.095**	10.71 ± 0.120**	10.73 ± 0.115**
*S. pyogenes*	10.98	10.43 ± 0.075**	10.45 ± 0.120**	10.50 ±.120**	11.67	10.84 ± 0.080**	10.90 ± 0.120**	10.92 ± 0.120**	11.25	10.98 ± 0.110**	10.99 ± 0.120**	11.01 ± 0.120**
*S. aureus* (MRSA)	10.76	10.21 ± 0.125**	10.23 ± 0.080**	10.24 ±.110**	11.00	10.37 ± 0.111**	10.40 ± 0.120**	10.42 ± 0.115**	11.11	10.56 ± 0.080**	10.59 ± 0.095**	10.63 ± 0.070**
*E. faecalis*	11.98	12.21 ± 0.057**	12.27 ± 0.120**	12.29 ±.080**	12.97	12.36 ± 0.110**	12.38 ± 0.080**	12.40 ± 0.070**	13.00	12.44 ± 0.110**	12.46 ± 0.120**	12.47 ± 0.115**
*E. aerogenes*	12.90	12.77 ± 0.111**	12.79 ± 0.110**	12.81 ±.095**	13.05	12.85 ± 0.120**	12.88 ± 0.080**	12.91 ± 0.120**	13.08	12.92 ± 0.120**	12.96 ± 0.120**	12.98 ± 0.110**
*E. coli*	14.86	14.45 ± 0.120**	14.57 ± 0.090**	14.66 ±.120**	14.95	14.55 ± 0.080**	14.62 ± 0.120**	14.69 ± 0.111**	15.00	14.74 ± 0.110**	14.76 ± 0.095**	14.79 ± 0.120**
*S. typhimurium*	12.30	12.05 ± 0.075**	12.16±.115**	12.20 ±.200**	12.98	12.16 ± 0.110**	12.18 ± 0.110**	12.19 ± 0.120**	13.07	12.26 ± 0.120**	12.29 ± 0.080**	12.31 ± 0.070**
*S. sonnei*	13.96	13.77 ± 0.125**	13.79 ± 0.120**	13.79 ±.110**	14.00	13.82 ± 0.115**	13.86 ± 0.115**	13.89 ± 0.080**	14.11	13.89 ± 0.120**	13.94 ± 0.115**	13.97 ± 0.110**
*P. vulgaris*	12.55	12.33 ± 0.120**	12.51 ± 0.080**	12.62 ± 0.120**	13.15	12.41 ± 0.095**	12.48 ± 0.070**	12.68 ± 0.125**	13.43	12.49 ± 0.080**	12.60 ± 0.111**	12.73 ± 0.172**
*P. mirabilis*	11.10	10.93 ± 0.120**	10.89 ± 0.304**	10.87 ± 0.115**	11.10	10.97 ± 0.080**	10.95 ± 0.111**	10.96 ± 0.172**	11.87	11.05 ± 0.080**	11.08 ± 0.120**	11.10±.120**
*K. pneumoniae*	13.00	12.87 ± 0.120**	12.85 ± 0.125**	12.80 ± 0.119**	13.30	12.91 ± 0.120**	12.94 ± 0.070**	12.95 ± 0.120**	13.20	12.97 ± 0.120**	13.00 ± 0.080**	13.02 ± 0.110**
*P. aeruginosa*	13.05	12.83 ± 0.080**	12.87 ± 0.115**	12.90 ± 0.080**	13.55	12.91 ± 0.115**	12.94 ± 0.120**	12.96 ± 0.080**	13.34	12.99 ± 0.110**	13.02 ± 0.120**	13.02 ± 0.080**
*C. jejuni*	13.96	13.30 ± 0.120**	13.34 ± 0.299**	13.37 ± 0.120**	14.18	13.38 ± 0.110**	13.40 ± 0.111**	13.42 ± 0.172**	14.09	13.45 ± 0.111**	13.48 ± 0.115**	13.50 ± 0.120**
*N. gonorrhoeae*	14.00	13.88 ± 0.150**	13.87 ± 0.120**	13.90 ± 0.070**	14.22	13.93 ± 0.080**	13.95 ± 0.125**	13.97 ± 0.070**	14.12	13.98 ± 0.125**	14.00 ± 0.120**	14.03 ± 0.115**
*H. influenzae*	13.96	13.55 ± 0.115**	13.65 ± 0.120**	13.66 ± 0.080**	14.66	13.62 ± 0.125**	13.71 ± 0.095**	13.75 ± 0.080**	14.1	13.69 ± 0.095**	13.78 ± 0.110**	13.82 ± 0.120**

**P ≤ 0.01; aValues are mean ± SD, SD = standard deviation.

**Table 5.** Phosphorus leakage (mmol·l^−1^) from bacterial cells after different periods of treatment with 50 µL of plant extracts.

Incubation periods/min												
	20				60				100			
	*CTAB (-ve+ C)*	*T. arabica*	*S. persica*	*T. arabica: S. persica*	*CTAB (-ve+ C)*	*T. arabica*	*S. persica*	*T. arabica: S. persica*	*CTAB (-ve+ C)*	*T. arabica*	*S. persica*	*T. arabica: S. persica*
control	-	6.10	6.10	6.10	-	6.31	6.31	6.31	-	6.40	6.40	6.40
*S. saparlyticus*	7.90	7.73 ± 0.021**	7.76 ± 0.021**	7.78 ± 0.026**	8.15	7.78 ± 0.032**	7.81 ± 0.032**	7.84 ± 0.032**	8.33	7.84 ± 0.017**	7.87 ± 0.023**	7.90 ± 0.026**
*S. pyogenes*	7.98	7.71 ± 0.007**	7.74 ± 0.032**	7.77 ± 0.017**	8.22	7.76 ± 0.015**	7.79 ± 0.017**	7.83 ± 0.015**	8.60	7.81 ± 0.029**	7.84 ± 0.026**	7.88± 0.095**
*S. aureus (MRSA)*	8.00	7.65 ± 0.023**	7.68 ± 0.021**	7.71 ± 0.010**	8.76	7.71 ± 0.020**	7.74 ± 0.015**	7.78± 0.017**	8.78	7.77± 0.095**	7.83 ± 0.017**	7.87 ± 0.012**
*E. mirabilis*	8.76	8.83 ± 0.017**	8.85 ± 0.020**	8.88 ± 0.087**	9.95	8.88 ± 0.026**	8.92 ± 0.032**	8.96 ± 0.026**	9.25	8.96 ± 0.010**	8.98 ± 0.040**	8.99 ± 0.017**
*E. aerogenes*	9.02	8.72 ± 0.020**	8.75 ± 0.015**	8.78 ± 0.010**	9.09	8.76 ± 0.011**	8.79 ± 0.032**	8.81 ± 0.015**	9.33	8.84 ± 0.011**	8.86 ± 0.029**	8.88 ± 0.040**
*E. coli*	10.88	10.43 ± 0.023**	10.47 ± 0.026**	10.49 ± 0.10**	11.00	10.48 ± 0.012**	10.55 ± 0.011**	10.58 ± 0.020**	11.15	10.61 ± 0.095**	10.63 ± 0.012**	10.66 ± 0.026**
*S. typhimurium*	9.11	8.62 ± 0.017**	8.64 ± 0.026**	8.67 ± 0.023**	9.19	8.68 ± 0.095**	8.70 ± 0.095**	8.73 ± 0.011**	9.67	8.75 ± 0.026**	8.77 ± 0.029**	8.79 ± 0.017**
*S. sonnei*	10.20	9.52 ± 0.026**	9.55 ± 0.023**	9.57 ± 00.17**	10.6	9.58 ± 0.012**	9.62 ± 0.026**	9.65 ± 0.095**	10.32	9.65 ± 0.026**	9.67±.029**	9.70 ± 0.011**
*P. vulgaris*	9.33	8.35 ± 0.017**	8.35 ± 0.029**	8.37 ± 00.17**	9.88	8.41 ± 0.026**	8.43 ± 0.026**	8.44 ± 0.010**	9.96	8.48 ± 0.020**	8.52 ± 0.015**	8.55 ± 0.026**
*P. mirobils*	7.98	7.32 ± 0.023**	7..35 ± 0.017**	7.36 ± 0.021**	8.48	7.38 ± 0.032**	7.40 ± 0.023**	7.42 ± 0..012**	8.54	7.45 ± 0.017**	7.45 ± 0.040**	7.46 ± 0.012**
*K. pneumoniae*	9.06	8.42 ± 0.026**	8.44 ± 0.095**	8.45 ± 0.021**	9.11	8.43 ± 0.017**	8.45 ± 0.015**	8.45 ± 0.020**	9.06	8.53 ± 0.011**	8.56 ± 0.026**	8.57 ± 0.029**
*P. aeruginosa*	9.14	8.45 ± 0.026**	8.47 ± 0.015**	8.47 ± 0.010**	9.56	8.49 ± 0.025**	8.53 ± 0.023**	8.56 ± 0.025**	9.22	8.55 ± 0.032**	8.58 ± 0.040**	8.58 ± 0.010**
*C. jejuni*	10.08	9.35 ± 0.030**	9.54 ± 0.023**	9.55 ±.017**	10.00	9.60 ± 0.011**	9.62 ± 0.015**	9.63 ± 0.011**	10.09	9.68 ± 0.095**	9.69 ± 0.029**	9.70 ± 0.020**
*N. gonohrae*	10.23	9.12 ± 0.015**	9.14 ± 0040**	9.17 ± 0.032**	10.00	9.21 ± 0.011**	9.24 ± 0.017**	9.25 ± 0.025**	10.12	9.30 ± 0.010**	9.32 ± 0.095**	9.34 ± 0.011**
*H. influenzae*	10.55	10.02 ± 0.032**	10.05 ± 0,025**	10.04 ± 0.036**	10.44	10.09 ± 0.015**	10.12 ± 0.015**	10.13 ± 0.095**	10.98	10.13 ± 0..011**	10.17 ± 0.029**	10.16 ± 0.012**

**P ≤ 0.01; aValues are mean ± SD, SD = standard deviation.

### 3.5. GC-MS analysis

#### 3.5.1. GC-MS analysis of *T. arabica* extract

**Figure 4.**
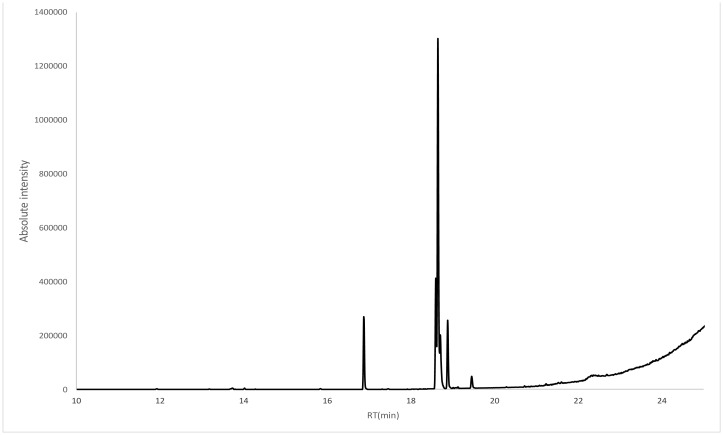
GC-MS analysis of the *T. arabica* extract.

GC was used to identify the components of the *T. arabica* extract. As shown in Table 6 and Figure 4, undecane-3,7-dimethyl was the first component observed, with a retention time of 6.23 min, while cyclodecasiloxane eicosamethyl was the last component observed, with a retention time of 28.58 min. According to the peak areas, the most abundant compound in the *T. arabica* extract was 6-octadecadienoic acid, methyl ester (Z), with a peak area of 2,502,182 m/z. The least abundant compound was naphthalene-1-sulfonic acid, 4-methoxy-,(2-adamantan-1-ylethyl) amide, with a peak area of 74,794 m/z.

#### 3.5.2. GC-MS analysis of *S. persica* extract

**Figure 5.**
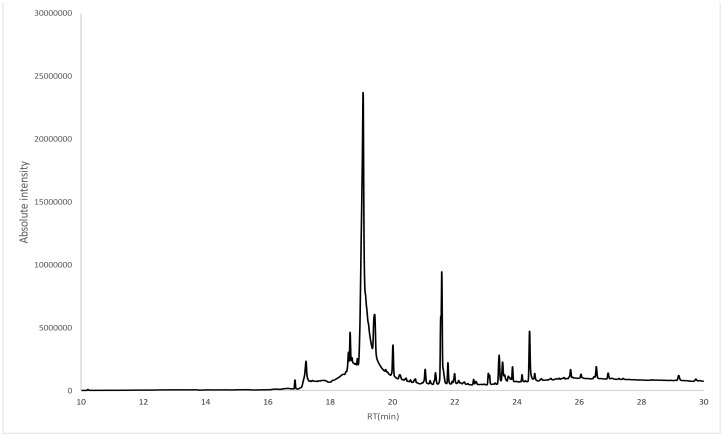
GC-MS analysis of the *S. persica* extract.

The components of the *S. persica* extract were also analyzed by GC (Table 7 and Figure 5). The first component observed was n-hexadecanoic acid, with a retention time of 17.207 min, while the last component observed was D:A-friedooleanan-3-ol, (3-alpha), with a retention time of 24.404 min. The most abundant compound in the *S. persica* extract was 6-octadecenoic acid, with a peak area of 165,478,838 m/z, while the least abundant compound was sulfurous acid cyclohexylmethyl pentadecyl ester, with a peak area of 1,962,094 m/z. The *S. persica* extract had a higher variety of compounds, which totaled fifteen, compared to *T. arabica*, which had ten compounds.

**Table 6.** GC-MS analysis of the *T. arabica* extract.

Peak#	RT(min)	Area	Height	A/H	Name
1	6.23	101082	27479	3.68	3,7-dimethylundecane
2	16.853	496357	268445	1.85	Hexadecanoic acid, methyl ester
3	18.575	773241	410523	1.88	11,14-Octadecadienoic acid, methyl ester
4	18.626	2502182	1E+06	1.93	6-Octadecenoic acid, methyl ester, (Z)-
5	18.683	434350	199550	2.18	9-Octadecenoic acid, methyl ester, (E)-
6	18.86	469366	252574	1.86	Methyl stearate
7	19.435	95315	43196	2.21	Phenol, 4,4′-(1-methylethylidene)bis-
8	28.025	74794	6585	11.4	Naphthalene-1-sulfonic acid, 4-methoxy-, (2-adamantan-1-yl) ethylamine
9	28.467	121618	11207	10.9	Silane, [(10-isodecyl)oxy]trimethyl-
10	28.58	211545	21639	9.78	Cyclodecasiloxane, eicosamethyl-

RT: retention time.

**Table 7.** GC-MS analysis of the *S. persica* extract.

Peak#	RT(min)	Area	Height	A/H	Name
1	17.207	3565026	1E + 06	2.78	n-Hexadecanoic acid
2	18.574	2744086	1E + 06	2.31	Methyl 10-trans,12-cis-octadecadienoate
3	18.627	4646460	2E + 06	1.89	9-Octadecenoic acid, methyl ester, (E)-
4	19.045	165478838	2E + 07	7.66	6-Octadecenoic acid
5	19.414	20759551	4E + 06	5.27	Phenol, 4,4′-(1-methylethylidene)bis-
6	20.005	5214162	2E + 06	2.2	Palmitoyl chloride
7	21.042	2125767	964904	2.2	Sulfurous acid, cyclohexylmethyl pentadecyl ester
8	21.542	10863490	5E + 06	2.1	9 12-octadecadienoic acid (z)- 2 3-dihydroxypropyl ester
9	21.578	19156345	9E + 06	2.19	Oleic anhydride
10	21.776	3286873	2E + 06	2.01	Octadecanoic acid, 2,3-dihydroxypropyl ester
11	23.076	3084877	885252	3.48	Terephthalic acid, but-3-enyl heptadecyl ester
12	23.424	6012053	2E + 06	2.78	6-Ethyl-3-trimethylsilyloxydecane
13	23.536	3046197	1E + 06	2.42	Urs-12-en-28-ol
14	23.855	1962094	1E + 06	1.84	Sulfurous acid, cyclohexylmethyl pentadecyl ester
15	24.404	11069113	4E + 06	2.79	D:A-Friedooleanan-3-ol, (3.alpha.)-

RT: retention time.

#### 3.5.3. GC-Ms analysis of *T. arabica*:*S. persica* extract

**Figure 6.**
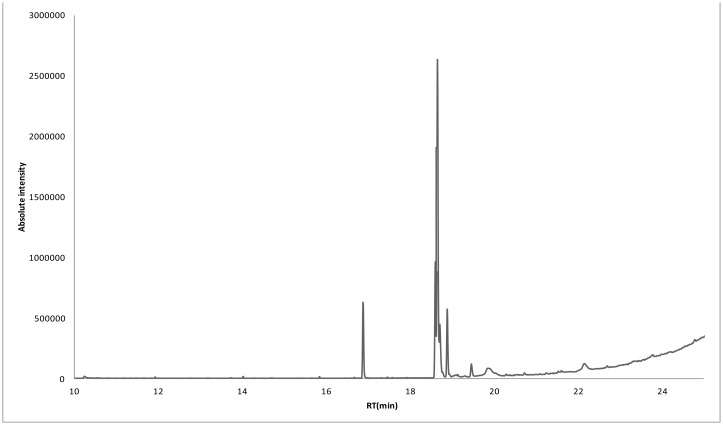
GC-MS analysis of the combined *T. arabica*:*S. persica* extract.

Table 8 and Figure 6 show the results of the analysis of *T. arabica*:*S. persica*. Some of the compounds from the individual plant extracts were retained in the mixture, but there were also new compounds formed. The newly formed compounds included undecane and methyl stearate. The first compound to be observed was undecane, with a retention time of 6.232 min and the last compound to be observed was octasiloxane-1,1,3,3,5,5,7,7,9,9,11,11,13,13,15,15-hexadecamethyl, with a retention time of 24.765 min. The most abundant compound in the extract mixture was 6-octadecenoic acid, methyl ester (Z), with a peak area of 5,131,843 m/z, while the least abundant compound was octasiloxane, with a peak area of 40,735 m/z.

**Table 8.** GC-MS analysis of the combined *T. arabica:S. persica* extract.

Peak#	RT (min)	Area	Height	A/H	Name
1	6.232	235833	56079	4.21	Undecane
2	16.853	1146651	622031	1.84	Hexadecanoic acid, methyl ester
3	18.575	1809625	958219	1.89	12,15-Octadecadienoic acid, methyl ester
4	18.627	5131843	3E+06	1.96	6-Octadecenoic acid, methyl ester, (Z)-
5	18.683	928724	438325	2.12	6-Octadecenoic acid, methyl ester, (Z)-
6	18.75	90871	42510	2.14	(1,2,2-trimethylbutyl)- Cyclohexane
7	18.86	1079535	559387	1.93	Methyl stearate
8	19.435	246014	102543	2.4	Phenol, 4,4′-(1-methylethylidene)bis-
9	19.827	359054	44559	8.06	Sulfurous acid, cyclohexylmethyl dodecyl ester
10	24.765	40735	17525	2.32	1,1,3,3,5,5,7,7,9,9,11,11,13,13,15,15-hexadecamethyl- Octasiloxane

RT: retention time.

## 4. Discussion

The Arabian Peninsula is characterized by unique environmental features, including a harsh ecology and limited water availability. As a result, plants growing in this region produce special compounds that give them the ability to survive in the harsh environment. Plants in this environment have been studied to investigate their potential bioactivities, including antimicrobial activity. The antimicrobial activities of *T. arabica* and *S. persica* have been shown to be due to bioactive compounds, including phenolic compounds, esters, organic acids, and oleic anhydride [25],[26]. Our results is agreed with the result of [42], Further, the result of *T. arabica* and *S. persica* against *S.* *aureus* (MRSA) showed a similar percentage to the result of [43] who used bacterial-produced TFnt against *S.* *aureus* 305 and Newman.

Both MIC and MBC values were determined against the tested bacteria, because, whereas the MIC value shows the lowest amount of antimicrobial agent required to inhibit growth, the MBC value shows the lowest amount of antimicrobial agent that results in the death of the microbes [44].

Measuring electrolyte leakage is a method of determining the stress response in intact cells. This phenomenon is unique among different species and cell types and can be activated by several factors. Therefore, an antimicrobial agent becomes more effective in its action against bacterial cells as time increases, up to the end of the incubation period. Leakage of potassium ions has detrimental effects on microorganisms, because potassium is a major structural and physiological component of microbial cells [45]. Potassium is transported across the membrane as part of the sodium-potassium pump. Leakage of phosphate ions is also destructive to microbial cells. Phosphorous has a range of functions in the cell that may be stalled in the presence of leakage. This may lead to the death of microbial cells. Phosphorous is used in cells to produce nucleic acid and for transport across membranes. Therefore, the leakage of phosphorous brings crucial microbial cell functions to a halt, leading to the destruction of the cells [46]. Unlike the leakage of potassium ions, the leakage of phosphorous decreases as time increases. The amount of phosphate leakage varied only slightly when cells are incubated with *T. arabica*, *S. persica*, or *T. arabica*:*S. persica* extracts. The antimicrobial activity of *T. arabica*, *S. persica*, and *T. arabica*:*S. persica* extracts, observed against the tested Gram-negative and Gram-positive bacteria, emphasize the importance of further investigations of these plants. MIC and MBC determinations showed that *T. arabica*, *S. persica*, and *T. arabica*:*S. persica* extracts were effective at inhibiting microbial growth. GC-MS results identified the active compounds in *T. arabica*, *S. persica*, and *T. arabica*:*S. persica* extracts. In conclusion, the plant extracts tested in this study and their bioactive compounds may represent promising candidates for new antibiotics, provided however that they are not toxic to human and animal.
